# Improving mental health referral readiness among providers encountering children from migrant backgrounds

**DOI:** 10.1016/j.jmh.2026.100452

**Published:** 2026-10-21

**Authors:** Karima Viksten Assel, Ana Hagström, Ester Gubi, Vanessa Place, Sofie Bäärnhielm, Cécile Rousseau, Christina Dalman, Anna-Clara Hollander, Johan Åhlén

**Affiliations:** a Department of Global Public Health, Karolinska Institute, Stockholm, Sweden; b Department of Learning, Informatics, Management and Ethics, Karolinska Institute, Stockholm, Sweden; c Centre for Epidemiology and Community Medicine, Region Stockholm, Stockholm, Sweden; d Center for Psychiatry Research, Department of Clinical Neuroscience, Karolinska Institute & Stockholm Health Care Services, Stockholm, Sweden; e Transcultural Centre, Region Stockholm, Stockholm, Sweden; f Division of Social and Cultural Psychiatry, McGill University, Montreal, Canada

**Keywords:** Migration, Access to care, Cross-cultural training, Public health intervention

## Abstract

**Introduction:**

Children from migrant backgrounds access mental health services to a lower extent than the majority population. Referrals from external providers are a potentially important route to care for these children. We evaluated a digitally delivered training program designed to strengthen referral readiness among providers working in community services, health care services and non-profit organizations in a Swedish municipality. Participants’ self-reported knowledge, sense of self-efficacy, and attitudes were evaluated.

**Methods:**

A longitudinal design was used, with a pre-training assessment and post assessments just after the training program and at a three-month follow-up. Data was self-reported by participants via electronic questionnaires.

**Results:**

189 respondents completed the pre-assessment and the assessment just after completion of the training, of which 65 completed the questionnaire at all three time points. Most respondents (n = 129, 68%) participated in training for community services. Although all self-reported knowledge and self-efficacy items were significantly strengthened post-intervention, knowledge of the local primary care based mental health services’ availability and organization was particularly improved. The effect decreased over time but remained improved at three-month follow-up compared to baseline.

**Conclusion:**

Uncertainty among referral agents about where to guide children in need of mental health support may contribute to preventing or delaying access to care for children from migrant backgrounds. Our results indicate that the training program has strengthened participants’ referral readiness, particularly their familiarity with local mental health services. Combined with other measures, improved referral readiness may be a valuable component in enhancing access to care for children from migrant backgrounds.

## Highlights

• Referral to mental health services may help reduce migrant children’s underutilization of services. However, there is limited understanding of effective methods to strengthen referring professionals’ knowledge of how to identify mental health problems in transcultural settings.
• This study shows that a training program for professionals and volunteers encountering children and families from migrant backgrounds, strengthened their referral readiness up to three months post-training.
• Professional training may thus be a promising avenue to enhance access to mental health services for children from migrant backgrounds.

## 1 Background

In 2015, before the introduction of more restrictive asylum legislation, the number of asylum-seeking children in Sweden peaked at over 70 000 applications, amongst whom around 50% were unaccompanied minors (Migrationsverket).

Children with experience of refugee migration are particularly affected by mental health disorders (Blackmore et al., 2020; Fazel et al., 2005). Yet a growing body of studies, several carried out in the Scandinavian care context, demonstrate that these children have a significantly lower use of mental health services compared to the majority population (Barghadouch et al., 2016; Berg et al., 2021; Gubi et al., 2022). While the mental health needs of non-refugee migrant children and youth are more uncertain, register-based studies indicate a lower service use also among this group as compared to their majority peers (Björkenstam et al., 2022; Gubi et al., 2022).

In 2025, the proportion of foreign-born children aged 0–17 in Sweden was 9% (Statistikmyndigheten). Given the importance of early intervention for mental health problems for children and youth (Budd et al., 2021; Werner-Seidler et al., 2021) the observed disparities in access to mental health care are of great concern.

There is currently limited knowledge of effective interventions to increase access to mental health services among children from migrant backgrounds. A scoping review on interventions to promote care-seeking among migrant populations for stigmatized disorders, including mental health conditions, was unable to draw any generalizable conclusions (Place, Nabb, Viksten Assel, et al., 2021). However, other research findings point to referral from external providers, such as primary care physicians and school staff, as important for children from migrant backgrounds’ access to mental health services. In Sweden, children from migrant backgrounds were more likely to come into contact with psychiatric services via referral compared to their peers (Ivert et al., 2011), and in Italy, school-based staff served as important “gate-openers” to mental health services for children with nationalities other than Italian (Pedrini et al., 2015). Moreover, in Canada, a dynamic collaboration between health care services and external actors has proven successful in building strong support networks around children from refugee backgrounds (Rousseau et al., 2013).

Given external referral agents’ important role in guiding patients to care, training programs to strengthen their ability to identify mental health problems in transcultural contexts, as well as knowledge of when and where to refer patients, is likely to facilitate access to care for migrant populations. Service providers’ limited transcultural competence has been identified as a barrier to care for children from migrant backgrounds (Place, Nabb, Gubi, et al., 2021). In contrast, an understanding of transcultural issues, an ability to recognize symptoms and to establish trust have been identified as important factors for successful referrals of refugees (Shannon et al., 2016).

There is limited knowledge as how to improve professionals’ mental health referral capacity in transcultural contexts. Cultural competency training (CCT), or cross-cultural training, has been demonstrated to strengthen various types of knowledge and skills among health care professionals (Beach et al., 2005) and mental health specialists (McDonald et al., 2021). However, in Sweden, as in many other settings, the responsibility for receiving newly arrived families is not limited to health care services but shared between several stakeholders, including community services that provide social services and schooling. In addition, non-profit organizations play an important role in supporting families’ resettlement.

Despite community professionals’ and volunteers’ position to guide migrant children and families to mental health care, the value of training these groups to increase their referral capacity, is not well understood. One previous study has, however, shown promising results: in an evaluation of a cross-cultural training program for mixed providers including social services and employment agencies, participants reported increased knowledge of how to identify mental health problems and of available care systems (Bäärnhielm et al., 2014).

With this study, we aimed to investigate weather training to diverse service providers working with migrant children in an urban Swedish municipality improved participants’ referral readiness. We specifically aimed to examine: 1. changes in participating providers’ referral readiness up to 3 months following a complex training program; 2. whether the outcome differed between providers from community services, health care and non-profit organizations; 3. whether providers’ previous experience of working with migrant families moderated the outcome.

Within this study, the outcome referral readiness was operationalized as self-reported knowledge to recognize mild to moderate mental health problems, knowledge of when to refer and of available mental health services. Referral readiness also included belief in own capacity to inquire about mental health issues, and attitudes towards working with families from migrant backgrounds, and towards health care.

The municipality where the training program was conducted is located in Stockholm County. In 2021, 28.7% of its population was foreign-born, to be compared with 20,0% in Sweden as a whole (Statistikmyndigheten (SCB)).

## 2 Methods

### 2.1 The primary care-based mental health service context

In the Stockholm Region, mental health problems of mild to moderate severity are typically managed within primary care based mental health services (PCMH). These problems range from subclinical symptoms to psychiatric conditions of moderate severity, and may include presentations such as anxiety, trouble sleeping, and behavioral difficulties. In line with regional guidelines, severity is assessed through an overall clinical evaluation, typically supported by instruments such as the Children’s Global Assessment Scale (CGAS), where scores above 60 generally indicate mild to moderate problems appropriate for primary care, while lower scores reflect more severe impairment requiring specialist services. PCMH services provide early and brief psychosocial interventions for children and adolescents with less severe symptoms and functional impairment, delivered by clinicians with generalist competencies.

In 2020, the municipality where the training was carried out hosted one of Stockholm County’s largest PCMH services for children and adolescents.

### 2.2 The training intervention

The training program was developed in collaboration with Transcultural Center, a regional center for migration and health, and was part of a larger public health project to facilitate care-seeking among children from migrant backgrounds (Place, Assel, et al., 2021).

In the Swedish context, the training program was innovative in that it sought to equip participants from health care services, community services and non-profit organizations with knowledge and self-efficacy to recognize mild to moderate mental health problems and refer children to the local PCMH clinic when appropriate. The training program included a core content of lectures covering the following concepts: migrant children’s mental health service use, psychosocial challenges of migration and displacement, “normal” stress reactions versus pathological responses in children and adolescents, the local primary care-based mental health services availability and organization and how to access local services (see Fig. 1). The presentations on service use discussed well-known barriers to care such as language barriers and stigma.

**Fig. 1:**
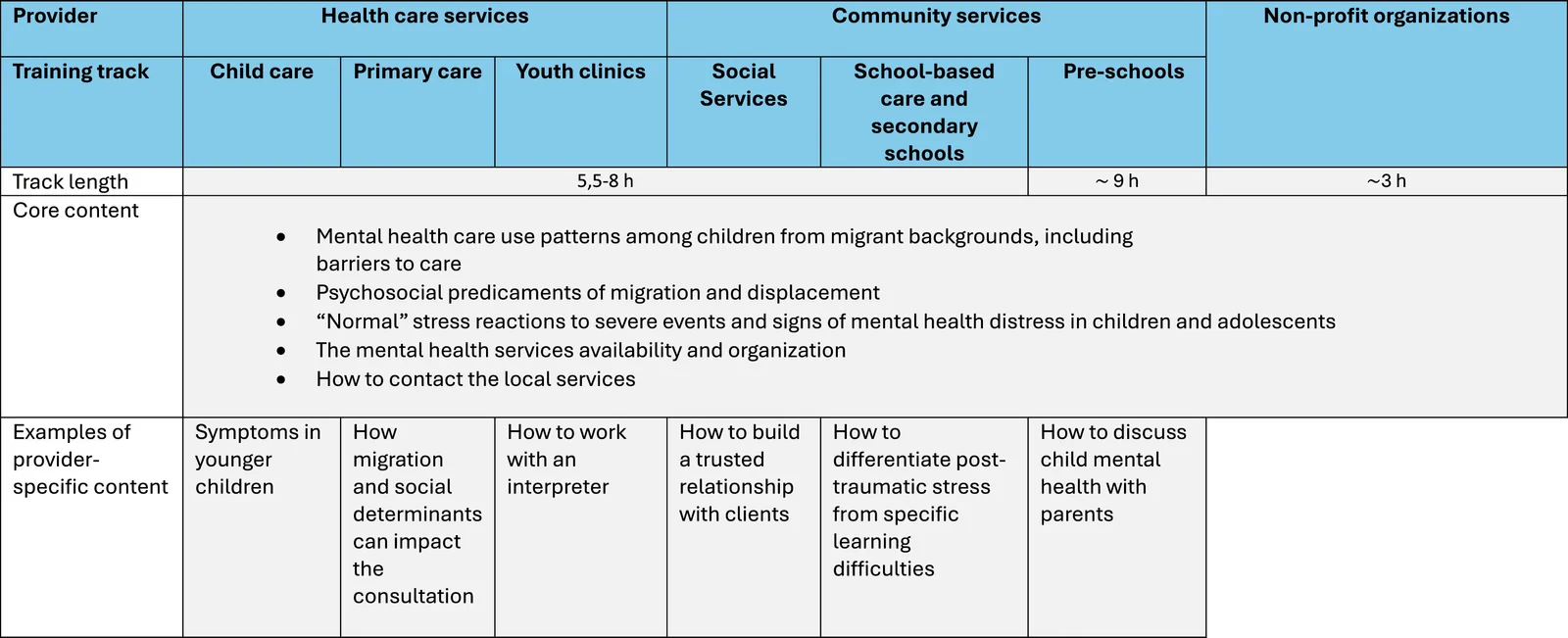
Schematic overview of the structure of the training program.

Given that severe mental health conditions requiring specialist psychiatric care fall outside the main scope of the referral pathway of the primary care based mental health services, these were therefore not emphasized in the training, although participants were informed about specialist psychiatric services to which more severe or urgent cases can be referred.

Besides the core content, the tracks for community and health care providers included provider-specific lectures, and self-reflection exercises on how to implement new knowledge in daily practice. These tracks also included interactive discussions about barriers to care, with the exception for the pre-recorded training for pre-school teachers, where this was not feasible. Interaction and questions from participants were encouraged. In some cases, the presenters used case discussions to exemplify. The educators included trainers from the regional competence center for migration and health, public mental health researchers, coordinators from the intervention municipality, and clinicians.

The development of the training intervention was informed by group interviews conducted with staff and volunteers from various providers about their training needs and preferences regarding format. The insights gained in the interviews, together with the Transcultural Center’s previous experiences of designing training, resulted in the development of seven separate needs-adapted curricular training tracks for units working in the same or nearby fields, as shown in Fig. 1 and described in the study protocol (Place, Assel, et al., 2021).

The participants were recruited from various sectors including social services, schools, child health and pediatric care, primary health care, and local non-profit organizations. Invitations were distributed to staff members via the head of units and key staff. Information was also advertised on the municipality’s internal website and distributed via the Transcultural Center. The recruitment was originally directed towards the intervention municipality’s geographical area, during the recruitment phase some training tracks were opened to a smaller number of interested staff from other municipalities. All training participation was voluntary.

The program was delivered over a six-month period between November 2020 and April 2021, except for one session which was held in December 2022. Due to the COVID-19 pandemic, all training was provided digitally. Despite the strained working conditions during the pandemic, most training was able to be carried out in a single day, except the training for primary care that had to be split into shorter sessions delivered over a 1–2-month period. Moreover, the training for pre-school teachers had to be converted into an online module with pre-recorded lectures that staff could work through at their own pace. In total 14 training sessions were carried out.

### 2.3 Study design and measures

A longitudinal design including a pre-assessment (T0), a post-assessment just after the training (T1), and a follow-up assessment 3 months after the training (T2), was used. Data was collected through electronic questionnaires. No previously validated questionnaires were deemed feasible for evaluating the training. Therefore, new questionnaires were designed for the study, inspired by instruments previously used to evaluate cross-cultural training (Bäärnhielm et al., 2014; McDonald et al., 2021). The items were not pilot tested but carefully reviewed by the research group which holds competence in transcultural mental health. The questionnaires were distributed before, or in connection with, the start of the training, just after completion of the training program, and 3 months after completion of the training.

The questionnaires covered themes such as the respondents’ professional role, their previous experience of encountering families from migrant backgrounds and their attitudes towards working with migrant families. The questionnaires also covered the respondents’ knowledge or self-efficacy in relation to topics such as psychosocial consequences of migration, signs of mental health distress, when to refer children, and the availability of mental health services. The respondents indicated on a Likert scale from 1–5 to what extent they agreed or disagreed with statements such as *“I have sufficient knowledge of signs of mental health distress in children”*. A shorter version of the questionnaire was developed for the volunteers. In this questionnaire, technical medical terminology was removed and six questions that were deemed irrelevant for the volunteer sector providers were omitted. For a summary of the items included in this study, see the Supplementary material. The questionnaires also included items to be used in a separate mixed methods process evaluation, such as on readiness for change. We estimate the completion time for each questionnaire to 10–15 min.

Within this study, a child from a “migrant background” was defined as a minor whose both parents have lived in Sweden <10 years, or a foreign-born unaccompanied minor who himself/herself has lived in Sweden for <10 years (Place, Assel, et al., 2021). This definition draws upon parents’ central role in children’s access to care, and upon findings suggesting underutilization of services among the adult migrant population during their first 10 years following resettlement (Hollander et al., 2020).

### 2.4 Study participants

The pre-training questionnaires were sent to 368 individuals, of which 274 respondents returned the questionnaire. Of the 274 respondents, 8 were excluded, for reasons explained in the flowchart (Fig. 2). Of the remaining 266 respondents, 189 also completed the questionnaire just after completion of the training, hereafter referred to as the study population. Of these, 65 respondents completed the questionnaire at all three time points. For more information on the study procedure, see the Supplementary material.

**Fig. 2:**
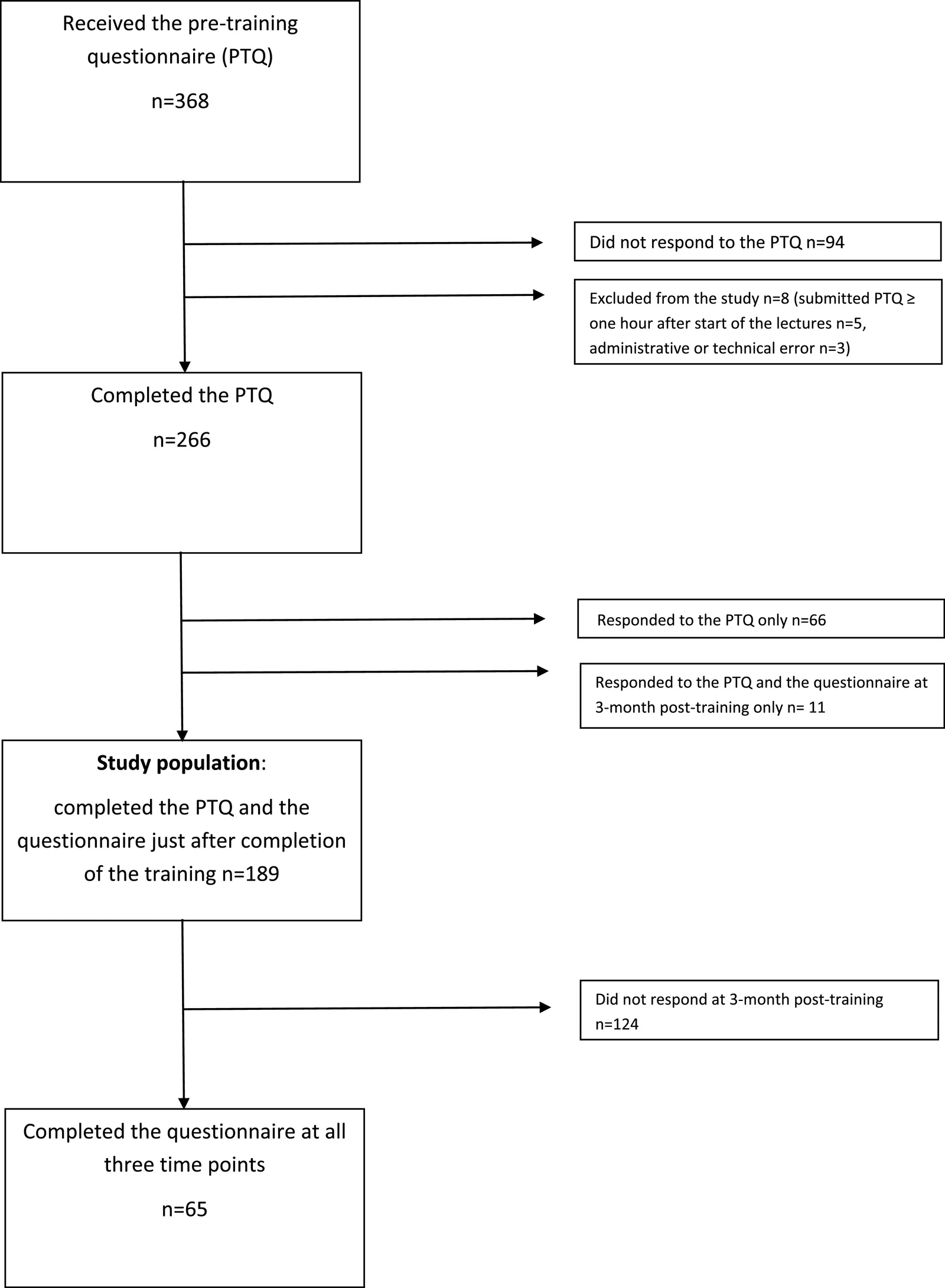
FLOWCHART.

### 2.5 Analyses

Statistical analyses were performed in the statistical software programs STATA 17.0, and JASP 0.18.3.0 (Love et al., 2019). Composite scores were created from all the items related to referral readiness based on results from a factor analysis. A scree plot and Very Simple Structure analysis supported a two-factor solution (See Supplementary Figure S1). The first factor included eight items reflecting attitudes and self-perceived knowledge related to migration and trauma and showed good internal consistency (Cronbach’s alpha .86). The second factor included seven items reflecting mental health-related knowledge and self-efficacy, also showing good internal consistency (Cronbach’s alpha .89). Composite scores were calculated for each factor at three time points using summed item scores. Repeated measures ANOVA was conducted to assess changes over time in both migration-related and mental health-related composite scores. To facilitate interpretation, standardized mean differences (Cohen's d) and corresponding 95% confidence intervals were calculated for pre-post changes. To examine the robustness of the findings, supplementary linear mixed-effects models were conducted for the two composite outcomes, including time as a fixed effect and participant as a random effect.

We also conducted Item-level analyses using repeated measures ANOVA. This allowed us to examine which specific items showed significant changes over time, providing a more detailed understanding of the training’s effects on attitudes, knowledge, and self-efficacy. To determine differences in training effects between the three main providers (community services, health care services and non-profit organizations), and between respondents’ previous experience level, we also used repeated measurement ANOVA. Missing data was deleted list-wise. Due to item-level missing data, the number of participants included in the item-specific analyses varied across outcomes and is reported for each analysis in the corresponding tables.

At baseline, respondents reported particularly high mean values for two items (interest in working with families from a migrant background (mean value: 4.6 out of 5, n = 196) and understanding of the psychosocial impact of migration (mean value: 4.9 out of 5 n = 179). As these remained high at the 3-month follow-up, these two items were excluded from the pre-and post-analyses regarding change. Also, due to the subgroup sample sizes being small (n = 4–9), respondents from non-profit organizations were excluded from the 3-month analysis and from the analysis of the training effects in relation to previous experience. Moreover, 6,9% (n = 13) of the respondents in the study population worked outside of the intervention municipality, and 16.4% (n = 31) had an undetermined workplace and were therefore potentially not familiar with the local health services. To make sure the result was not driven by these groups, a sensitivity analysis was performed for the item covering knowledge of the local primary-care based services.

### 2.6 Participant involvement statement

The content and format of the training program was informed by interviews with staff and volunteers about their training needs and preferences. Patients or members of the public were not involved in the study design or in the choice of measures.

## 3 Results

### 3.1 Study participants’ experience of working with families

68.3% (n = 129) of the study population attended training directed towards community services, whereas 22.2% (n = 42) took part in training for health care providers, and 9.5% (n = 18) in training for non-profit organizations. Between 77.8% and 85.7% of the respondents reported having encountered children or families from migrant backgrounds within the previous month, whereas between 22.2% and 45.2% had referred children or families (with or without migrant backgrounds). Between 64.3% and 65.1% of the respondents from community services and health care services reported having worked with an interpreter within the previous month. Further information on the study population’s experience is presented in Table 1.

**Table 1:** Study participants’ experiences of working with families.

Experiences	Training program’s target group		
	Community services n = 129 (68.3%)	Health care services n = 42 (22.2%)	Non-profit organisations n = 18 (9.5%)
Is in contact with children in their professional capacity.	91⁎(76.5%)	38 (90.5%)	n/a
Has encountered children and/or families from a migration background within the previous month.	107 (82.9%)	36 (85.7%)	14 (77.8%)
Has worked with an interpreter within the previous month.	84 (65.1%)	27 (64.3%)	n/a
Has referred children or families for mental health distress within the previous month.	45 (34.9%)	19 (45.2%)	4 (22.2%)
Holds a manager’s role.	7* (5.9%)	1 (2.4%)	n/a
Works unpaid.	n/a	n/a	4 (22.2%)
Reported workplace matches the training tracks’ target group.	119 (92.2%)	33 (78.6%)	n/a

⁎ Missing data n = 10. n/a= did not receive the question.

### 3.2 Changes in migration-related and mental health-related composite scores

A repeated measures ANOVA on the migrant related composite scores from pre- and post-training, showed increased mean scores from 27.2 (SD = 6.2) before the training to 33.1 (SD = 4.1) after the training. The analysis revealed a statistically significant effect of time, F(1, 151) = 162.6, p < .001. The standardized mean difference (SMD) for the pre-post change was 1.03 (95% CI [0.84, 1.23]). Similarly, mental health-related composite score increased from 21.9 (SD = 6.1) pre-training to 29.5 (SD = 3.7) post-training. The repeated measures ANOVA showed a significant effect of time, F(1, 153) = 271.8, p < .001. The pre-post change corresponded to an SMD of 1.33 (95% CI [1.11, 1.54]). Results from the mixed-effects models were consistent with those from the repeated-measures ANOVAs, showing significant improvements over time for both migration-related, t = 12.93, p < .001, and mental health-related composite scores, t = 16,8 p < .001.

Follow-up data were only available for a smaller subset of participants (n = 65). For migration-related composite scores, the mean score at follow-up was 32.3 (SD = 5.3), showing a marginal decrease compared to post-training. A repeated measures ANOVA across all three time points (pre, post, follow-up) still showed a significant effect of time, F (2, 205) = 96.2,p < .001, indicating that the training effects were largely sustained. The SMD between baseline and follow-up was 0.93 (95% CI [0.60, 1.26]).

For mental health-related composite scores, the follow-up mean was 27.2 (SD = 5.4), also slightly lower than the post-training score. The repeated measures ANOVA revealed a significant effect of time, F (2, 208) = 159.2,p < .001. The SMD between baseline and follow-up was 1.12 (95% CI [0.78, 1.46]). Again, mixed-effects models were consistent with those from the repeated-measures ANOVAs, showing significant improvements between baseline and follow-up for both migration-related, t = 7.61, p < .001, and mental health-related composite scores, t = 8.33 p < .001. These results suggest that while some decline occurred over time, participants retained a substantial portion of the knowledge gained in training. Due to the limited number of follow-up responses, these findings should be interpreted with caution.

Attrition analyses comparing participants retained at follow-up with those lost to follow-up revealed no significant differences in service setting, recent contact with migrant families, or recent mental health referrals. The groups also did not differ significantly in baseline migration-related knowledge or baseline mental health-related knowledge (Supplementary Table S6). Furthermore, the interaction between attrition status and time (pre-post) was not statistically significant, indicating that patterns of change over time did not differ between participants retained and lost to follow-up, *F*(1, 150) = 0.21, *p* = .65, and *F*(1, 152) < 0.01, *p* = .96, for migration-related and mental health-related knowledge, respectively.

### 3.3 Item-based changes in attitudes, knowledge and self-efficacy

As illustrated in Table 2, all knowledge and self-efficacy items were significantly strengthened directly after completion of the training programs. Knowledge of the local primary-care based mental health services was particularly improved. Although the effect weakened over time for some items, the increase was still present at 3-month follow-up for all knowledge and self-efficacy items compared to baseline, as shown in Table 3. There were no significant changes in attitudes following completion of the training program.

**Table 2:** Mean values and changes in attitudes, knowledge and self-efficacy (T0 and T1).

Questionnaire item (shortened)^1^	T0 mean (Sd)	T1 mean (Sd)	Mean difference	T0-T1	
				F -value	p-value
**Item asked to all providers**					
Attitude towards whether it is challenging to work with children and families from migrant backgrounds n = 178	2.9 (1.3)	3.0 (1.3)	0.1	0.8	0.384
Attitude towards whether healthcare for children in Sweden is equitable. n = 160	3.3 (1.5)	3.5 (1.4)	0.1	1.9	0.171
Sufficient knowledge of signs of mental health distress. n = 185	3.3 (1.1)	4.0 (0.7)	0.8	101.8	**<0.001**
Sufficient knowledge of how to work to prevent mental health distress. n = 183	3.1 (1.1)	3.9 (0.8)	0.8	83.8	**<0.001**
Sufficient knowledge of how the migration process can impact living conditions. n = 186	3.1 (1.2)	4.3 (0.7)	1.1	200.5	**<0.001**
Sufficient knowledge of how the migration process can impact psychosocial well-being. n = 187	3.1 (1.1)	4.3 (0.7)	1.1	196.9	**<0.001**
Sufficient knowledge of how traumatic events can impact mental health. n = 188	3.4 (1.1)	4.3 (0.7)	0.9	127.9	**<0.001**
Sufficient knowledge of potential barriers to care. n = 183	3.3 (1.1)	4.3 (0.7)	1.1	159.4	**<0.001**
**Item asked to community and health care services only**					
Confidence in own ability to know how and when to inquire about mental health distress. n = 167	4.0 (0.9)	4.3 (0.7)	0.3	22.1	**<0.001**
Confidence in own ability to know how and when to inquire about traumatic events. n = 165	3.6 (1.0)	4.0 (0.8)	0.4	33.4	**<0.001**
Sufficient knowledge of how and when to bring up mental health and trauma. n = 167	3.2 (1.1)	4.0 (0.7)	0.8	101.1	**<0.001**
Sufficient knowledge of how cultural and social factors may impact communication. n = 169	3.6 (1.0)	4.4 (0.6)	0.8	93.0	**<0.001**
Sufficient knowledge of when to guide/refer children to health care. n = 165	3.4 (1.1)	4.3 (0.7)	0.9	110.2	**<0.001**
Sufficient knowledge of when to guide/refer children to primary-care or child psychiatry. n = 165	3.5 (1.1)	4.3 (0.7)	0.9	106.4	**<0.001**
Sufficient knowledge of the collaboration between the municipality and the health care services. n = 165	2.9 (1.2)	4.1 (0.8)	1.2	153.2	**<0.001**
Sufficient knowledge of the services offered at the local primary-care based mental health services n = 166	2.6 (1.2)	4.6 (0.7)	2.0	381.2	**<0.001**
**Item asked to non-profit-organizations only**					
Sufficient knowledge of how and when to discuss mental health distress with children and families. n = 18	2.7 (1.2)	3.3 (1.0)	0.7	7.6	**0.014**
Sufficient knowledge of where minors with mental health distress can go for support within the health care system. n = 18	2.4 (1.1)	3.8 (0.8)	1.4	41.3	**<0.001**
Sufficient knowledge of services and support offered at the local primary-care based mental health services. n = 17	2.1 (1.1)	4.1 (1.0)	2.1	42.8	**<0.001**

Measure points: before the start of the training program (T0) and just after completion of the training program (T1).

Fully disagree = 1, fully agree = 5

^1^See the supplementary material for full questions.

**Table 3:** Mean values and changes in attitudes, knowledge and self-efficacy (T0, T1 and T2).

Questionnaire item summary (shortened)^1^	T0 mean (Sd)	T1 mean (Sd)	T2 mean (Sd)	T0-T2		POST HOC T0-T2`⁎⁎		
				F-value	P-value	T0-T1	T0-T2	T1-T2
						Diff, p-value	Diff, p-value	Diff, p-value
**Item asked to all providers**								
Attitude towards whether it is challenging to work with children and families from migrant backgrounds. n = 60	2.7 (1.4)	2.8 (1.4)	2.4 (1.3)	2.7	0.070			
Attitude towards whether healthcare in Sweden is equitable. n = 56	3.5 (1.4)	3.4 (1.4)	3.2 (1.3)	2.7*	0.082*			
Sufficient knowledge of signs of mental health distress. n = 64	3.3 (1.1)	4.1 (0.7)	3.8 (0.9)	26.3	**<0.001**	0.8 **<0.001**	0.6 **<0.001**	−0.3 **0.031**
Sufficient knowledge of how to work to prevent mental health distress. n = 63	3.1 (1.2)	3.9 (0.8)	3.7 (0.9)	21.2	**<0.001**	0.9 **<0.001**	0.7 **<0.001**	−0.2 0.170
Sufficient knowledge of how the migration process can impact living conditions. n = 62	3.2 (1.2)	4.4 (0.6)	4.0 (1.0)	49.0⁎	**<0.001**⁎	1.2 **<0.001**	0.8 **<0.001**	−0.4 **0.002**
Sufficient knowledge of how the migration process can impact psychosocial well-being. n = 62	3.2 (1.2)	4.4 (0.6)	4.0 (1.0)	46.0⁎	**<0.001**⁎	1.2 **<** **0.001**	0.8 **<0.001**	−0.4 **0.002**
Sufficient knowledge of how traumatic events can impact mental health. n = 63	3.4 (1.1)	4.4 (0.6)	4.1 (1.0)	33.1	**<0.001**	0.9 **<0.001**	0.6 **<0.001**	−0.3 **0.011**
Sufficient knowledge of potential barriers to care. n = 63	3.3 (1.0)	4.4 (0.7)	4.0 (0.9)	40.3⁎	<0.001⁎	1.1 **<0.001**	0.7 **<0.001**	−0.3 **0.005**
**Item asked to community services and health care only**								
Confidence in own ability to know how and when to inquire about mental health distress. n = 59	3.8 (1.0)	4.2 (0.7)	4.2 (0.9)	8.3*	**<0.001**⁎	0.4 **<0.001**	0.4 **0.005**	−0.1 0.461
Confidence in own ability to know how and when to inquire about traumatic events. n = 56	3.6 (1.0)	4.1 (0.8)	3.9 (1.0)	7.7	**<0.001**	0.5 **<0.001**	0.3 **0.032**	−0.2 0.153
Sufficient knowledge of how and when to bring up mental health distress and trauma. n = 58	3.2 (1.1)	4.0 (0.8)	3.8 (1.0)	20.0	**<0.001**	0.8 **<0.001**	0.6 **<0.001**	−0.2 0.106
Sufficient knowledge of how cultural and social factors can impact communication. n = 58	3.6 (0.9)	4.4 (0.6)	4.3 (0.7)	28.7*	**<0.001**⁎	0.8 **<0.001**	0.7 **<0.001**	−0.1 0.465
Sufficient knowledge of when to guide/refer children to health care. n = 58	3.5 (1.1)	4.3 (0.7)	4.0 (0.9)	30.0	**<0.001**	0.8 **<0.001**	0.5 **<0.001**	−0.3 **0.007**
Sufficient knowledge of when to guide/refer children to primary-care or child psychiatry. n = 58	3.6 (1.2)	4.4 (0.7)	4.0 (0.9)	22.4	**<0.001**	0.8 **<0.001**	0.4 **0.002**	−0.4 **0.002**
Sufficient knowledge of the collaboration between the municipality and the health care services. n = 58	3.0 (1.1)	4.2 (0.8)	3.8 (1.0)	38.3*	**<0.001**⁎	1.2 **<0.001**	0.8 **<0.001**	−0.4 **0.003**
Sufficient knowledge of the services offered at the local primary-care based mental health services. n = 58	2.8 (1.3)	4.8 (0.6)	4.0 (1.1)	99.3*	**<0.001**⁎	2.0 **<0.001**	1.3 **<0.001**	−0.7 **<0.001**

Measure points: before the start of the training program (T0), just after completion of the training program (T1) and 3 months after completion of the training (T2). Fully disagree = 1, fully agree = 5.

⁎ Sphericity corrected with Greenhouse-Geisser.

⁎⁎ All post-hoc p-values corrected with Holm for a family of three ^1^See the supplementary material for full questions.

### 3.4 Differences between providers

Directly after completion of the training program, for most items there was no statistically significant difference in training effect between community services, health care services and non-profit organizations, as shown in Supplementary Table S2. However, for five knowledge items, such as when to guide children to health care, respondents from community services reported a significantly larger increase, as compared to respondents from other providers.

### 3.5 Respondents’ previous level of experience

Just after completion of the training, there was no statistically significant difference in training effect between respondents who in the previous month had often, sometimes, or never met with children and families from migrant backgrounds, as shown in Supplementary Table S3.

### 3.6 Sensitivity analyses

The statistically significant change in knowledge of the local primary care-based services just after the training program and at 3-month follow-up as compared to baseline, remained when respondents working outside of the intervention municipality, or with uncertain location of workplace, were excluded. For the results, see Supplementary Tables S4 and S5.

## 4 Discussion

This study was a pragmatic attempt to explore whether a complex training intervention for diverse providers in a Swedish municipality changed participants’ referral readiness. Our results suggest that the program strengthened participants’ knowledge and self-efficacy, while attitudes remained unchanged. The training effect decreased over time but remained at three-month follow-up compared to baseline.

Referral from external community providers is likely important for migrant children’s access to care (Ivert et al., 2011; Pedrini et al., 2015). This study suggest that this type of needs-adapted training program may be a valuable tool to improve staff and volunteers’ knowledge of how to identify mental health distress, when to refer and of available local mental health services. In combination with other actions, training to improve referral readiness may be a useful component in increasing access to mental health services for children from migrant backgrounds. In Sweden, foreign-born parents identified uncertainty about where to seek help and language barriers as particularly important barriers to accessing mental health services for their children (Gubi et al., 2024).

Cultural competency training (CCT) has been shown to enhance health professionals’ knowledge about cultural factors, (Beach et al., 2005), while the effect on patient outcomes has been small or lacking (Horvat et al., 2014; Vella et al., 2022). Besides knowledge, changes at the organizational level are often needed for employees to be able to implement new cultural competencies (Truong et al., 2014). However, the training program in this study differed from typical CCT in that it placed greater emphasis on the organization of local mental health services and referral pathways. As such, it may have had more similarities with general mental health literacy training, although focused on migration related mental health. Previous studies indicate that mental health literacy training for staff may influence referrals to mental health services, but the exact mechanisms are not clear. In a study with school staff, children and youth who were referred to mental health services by staff who had participated in mental health literacy training had different clinical profiles and treatment outcomes from those referred by untrained staff, suggesting a positive change in the quality of referrals (Baxter et al., 2022). Similarly, another study found that primary care physicians made more adequate referrals after having participated in child mental health training (McCaffrey et al., 2017). It remains to verify if the changes in knowledge and self-efficacy observed in this study, indicating strengthened referral readiness, translate into changes in actual referral behaviours.

Importantly, participants’ familiarity with local mental health services, was particularly increased post-intervention. This finding suggests the presence of silos within a fragmented system of child and adolescent services, where providers are unaware of each other’s role and availability. Reluctance or mistrust towards mental health services (Colucci et al., 2015; Place, Nabb, Gubi, et al., 2021) and uncertainty about where to seek help (Gubi et al., 2024) are well-described barriers to care for children and youth from migrant backgrounds. Less attention has been given to the uncertainty among staff about where to direct children in need of support, although limited communication between providers have previously been identified as an important barrier to care (Colucci et al., 2015). Our results demonstrate the value of making explicit how local providers are organized in future training with the objective of increasing access to mental health services, as this knowledge may not be widely shared amongst community professionals. The findings from this study call for a strengthening of structures that enable information-sharing and collaboration between community providers and health care services to ensure access to care for vulnerable groups.

Participant knowledge of the social predicaments of migration was another area strengthened by the training program. The importance of addressing social factors to overcome barriers to care has been increasingly recognized, and a structural competency approach has been integrated in the training of psychiatry residents to equip clinicians with skills to address social determinants of health (Hansen et al., 2018). It would have been interesting to know whether the training participants’ awareness of the availability of social services was strengthened to the same extent as that of the mental health services following the training program, but unfortunately no such question was asked in the questionnaire.

Another key finding of this study is the respondents’ high interest in working with families from migrant backgrounds at base line (mean value: 4.6 out of 5). It is difficult to know if this level of interest is related to the intervention municipality’s context and/or to the period when the study was carried out. In 2015, Sweden received a large number of asylum-seekers (Riksrevisionen, 2017) with considerable media and public interest in migration and migrant rights issues as a consequence in the following years. Since 2016, more restrictive immigration legislation has been introduced with subsequent reduction in overall immigration, and popular support for anti-immigration policies has increased. Besides the wider societal context, the local municipality context may have influenced participant’s engagement.

In contrast to the observed changes in participant knowledge and self-efficacy, attitudes working with migrant families and towards health care services remained unchanged. This may reflect the fact that changing attitudes is a process that often takes time, but also that the time restrained format may be less suitable for deeper reflections around subjects such as trust and discrimination.

The training was delivered using different formats, including same-day sessions, split sessions, and pre-recorded modules, reflecting the pragmatic implementation context during the COVID-19 pandemic. We explored whether training effects differed across provider groups (community services, health care, and non-profit organizations), which largely corresponded to different training tracks with somewhat varied delivery formats. The fact that no substantial differences in outcome were observed across provider groups could indicate that the tailored format was generally successful in meeting the diverse needs of participants. Linking training content to participants’ needs and prior knowledge has been proposed to be among factors supporting in-depth learning (Biggs and Tang, 2011). However, the subgroup analyses were likely underpowered, particularly given the small number of participants in some groups (e.g., non-profit organizations n = 18). Therefore, the absence of statistically significant differences between groups should be interpreted cautiously.

It is likely that the knowledge gained via the training will diminish with time, although the information could also be consolidated when used in daily practice. Repeated training sessions would likely have better sustained the effect of training but can be potentially challenging to implement in participant groups with little time to set aside for training. Digital delivery could facilitate booster sessions, given that participants can join from any location, although interactivity may be reduced compared to face-face training interaction opportunities. Another factor that may affect implementation of new practices, is the commitment of managers (Reichenpfader et al., 2015). Although a high degree of engagement from key stakeholders was present in the planning phase, very few of the participants in the training program held a manager’s role. Stronger manger participation would likely have been beneficial to sustain implementation of new routines.

Finally, notwithstanding the important role of referring agents in guiding families to adequate services, professional referrals may be linked to coercion to a higher extent than self-referral (Ivert et al., 2011). Thus, health care systems also need to be designed so that patients independently can navigate the health care system and reach care on their own. Moreover, improved access to services, although beneficial, does not ensure social and cultural adaptation of services, nor protection against discrimination.

### 4.1 Strengths and limitations

The main strength of the study was the attempt to address a well-known disparity in access to care for a vulnerable group, by evaluating a needs-adapted training program in which staff and volunteers influenced the content. Another strength is the follow-up of the outcome measures over time. The number of non-responders was low at the first follow-up just after the program but increased at second follow-up at 3-month after completion of program.

The limitations of this study relate first to the methodology. The pre-post design without using a control group could mean that the effects are overestimated. Importantly, the outcomes assessed in this study were self-reported and reflect the participants’ perceived knowledge and self-efficacy, rather than actual referral behavior or changes in access to mental health services for children. Therefore, it remains unknown whether the observed improvements in referral readiness translated into changes in clinical practice or improved service utilization. Another limitation is that the sample size at 3 months follow-up was small (n = 65). The attrition rate may have been influenced by the Covid-19 pandemic, the fact that there was no financial compensation for participating, and our limited opportunity to remind participants to respond (by email only).

The fact that a large majority of the respondents had met with children or families from migrant backgrounds in the previous month could indicate that the training program reached the intended participants. However, the high interest among the respondents may indicate a potential bias in that the training attracted a selected group of already engaged staff, while other providers, who also encounter migrant families, did not take part. The study may well have produced different results in another group of participants. Moreover, data on attendance, participant engagement, and completion were not systematically collected as part of this pragmatic evaluation. Finally, the use of previously validated questionnaires would have increased the reliability of the results.

## 5 Conclusion

The findings of this study show that a needs-adapted training program for community services, health care services and non-profit organizations enhanced participants’ referral readiness, by strengthening their self-reported knowledge and self-efficacy. Strengthened knowledge of how to identify mental health problems and when to refer may, in turn, contribute to overcoming barriers to mental health services for children from migrant backgrounds. Our results encourage designers of similar training programs to incorporate information on local mental health providers’ availability and organization, as this type of knowledge may not be widely known. Further research is needed to evaluate the effectiveness of training on actual referral behaviors. Future studies may also wish to address the cultural sensitivity and inclusiveness of services from parents and youth perspectives, in partnership with community stakeholders.

## Ethics declaration

Written informed consent to take part in the study and to publish the article has been obtained from all participants or their legal representatives. The privacy rights of participants have been observed.

This study was performed in compliance with relevant laws, regulatory frameworks and guidelines where the research took place. This study was approved by the Swedish Ethical Review Board . (Approval no 2019-06275, 2020-03641, 2020-03642.)

## Funding

This study was funded by the Swedish Research Council.

## CRediT authorship contribution statement

**Karima Viksten Assel:** Writing – original draft, Methodology, Formal analysis, Data curation, Conceptualization, Writing – review & editing. **Ana Hagström:** Writing – review & editing, Methodology, Data curation, Conceptualization. **Ester Gubi:** Writing – review & editing, Writing – original draft. **Vanessa Place:** Writing – review & editing, Writing – original draft. **Sofie Bäärnhielm:** Writing – review & editing, Resources. **Cécile Rousseau:** Writing – review & editing, Writing – original draft. **Christina Dalman:** Writing – review & editing, Resources, Funding acquisition, Conceptualization. **Anna-Clara Hollander:** Writing – review & editing, Methodology, Conceptualization. **Johan Åhlén:** Writing – review & editing, Supervision, Methodology, Formal analysis, Data curation, Conceptualization.

## Declaration of competing interest

The authors declare that they have no known competing financial interests or personal relationships that could have appeared to influence the work reported in this paper.

## Data Availability

The underlying data may be made available upon request and after ensuring compliance with relevant legislation and KI guidelines.
