# Supplementary material for: Improving mental health referral readiness among providers encountering children from migrant backgrounds
Source: J Migr Health. 2026 Oct 21;14:100452. doi: 10.1016/j.jmh.2026.100452 (PMC13744873; doi:10.1016/j.jmh.2026.100452)
Supplement: Supplementary file 1 [file mmc1.docx]

**Supplementary material**

Content:

- Supplementary Table S1
- Supplementary Figure S1
- Supplementary Tables S2-5
- Supplementary Table S6
- Questionnaire items on knowledge, attitudes and self-efficacy
- Additional information on the study procedure

**Supplementary Table S1.** Exploratory Factor Analysis using a two-factor solution of the survey items

| **Survey Items** | **Factor 1** | **Factor 2** |
| --- | --- | --- |
| Attitude towards whether it is challenging to work with children and families from migrant backgrounds |  | -.46 |
| Attitude towards whether healthcare for children in Sweden is equitable |  |  |
| Sufficient knowledge of signs of mental health distress | .69 |  |
| Sufficient knowledge of how to work to prevent mental health distress | .69 |  |
| Sufficient knowledge of how the migration process can impact living conditions |  | .90 |
| Sufficient knowledge of how the migration process can impact psychosocial well-being |  | .95 |
| Sufficient knowledge of how traumatic events can impact mental health. |  | .75 |
| Sufficient knowledge of potential barriers to care |  | .58 |
| Confidence in own ability to know how and when to inquire about mental health distress | .64 |  |
| Confidence in own ability to know how and when to inquire about traumatic events |  | .49 |
| Sufficient knowledge of how and when to bring up mental health and trauma |  | .38 |
| Sufficient knowledge of how cultural and social factors may impact communication |  | .64 |
| Sufficient knowledge of when to guide/refer children to health care | .92 |  |
| Sufficient knowledge of when to guide/refer children to primary-care or child psychiatry | .83 |  |
| Sufficient knowledge of the collaboration between the municipality and the health care services. | .65 |  |
| Sufficient knowledge of the services offered at the local primary-care based mental health services | .59 |  |

*Notes:* Loadings below .3 are excluded from the table.

**Supplementary Figure S1.** Scree plot of Survey items

**Supplementary Table S2.** Mean values and changes in attitudes, knowledge and self-efficacy (T0 and T1), by provider.

| **Questionnaire item summary (shortened)^1^** | **Training track to community services** | | **Training track to health care services** | | **Training track to non-profit organizations** | | **Interaction provider*time** |
| --- | --- | --- | --- | --- | --- | --- | --- |
|  | T0 mean (Sd) | T1 mean (Sd) | T0 mean (Sd) | T1 mean (Sd) | T0 mean (Sd) | T1 mean (Sd) | **P-value** |
| **Item asked to all providers** |  |  |  |  |  |  |  |
| Attitude towards whether it is challenging to work with children and families from migrant backgrounds. | 2.8 (1.3)  n=118 | 2.7 (1.3)  n=118 | 3.4 (1.1)  n=42 | 3.8 (0.9)  n=42 | 2.4 (1.4)  n=18 | 2.6 (1.3)  n=18 | 0.374 |
| Attitude towards whether healthcare in Sweden is equitable. | 3.4 (1.5)  n=103 | 3.6 (1.4)  n=103 | 3.4 (1.4)  n=42 | 3.4 (1.4)  n=42 | 2.7 (1.6)  n=15 | 2.9 (1.5)  n=15 | 0.504 |
| Sufficient knowledge of signs of mental health distress. | 3.2 (1.1) n=126 | 4.1 (0.7)  n=126 | 3.7 (0.9)  n=41 | 4.2 (0.6)  n=41 | 2.7(1.1)  n=18 | 3.6 (1.0)  n=18 | 0.125 |
| Sufficient knowledge of how I can work to prevent mental health distress. | 3.1 (1.1)  n=124 | 3.9 (0.8)  n=124 | 3.5 (1.0)  n=41 | 3.9 (0.8)  n=41 | 2.8 (1.3)  n=18 | 3.7 (0.7)  n=18 | 0.081 |
| Sufficient knowledge of how the migration process can impact living conditions. | 3.1 (1.1)  n=128 | 4.4 (0.7)  n=128 | 3.0 (1.0)  n=41 | 4.1 (0.7)  n=41 | 3.2 (1.6)  n=17 | 3.9 (0.7)  n=17 | 0.206 |
| Sufficient knowledge of how the migration process can impact well-being. | 3.2 (1.1)  n=129 | 4.4 (0.7)  n=129 | 3.0 (1.0)  n=41 | 4.1 (0.7)  n=41 | 3.1 (1.5)  n=17 | 3.7 (1.0)  n=17 | 0.096 |
| Sufficient knowledge of how traumatic events can impact mental health. | 3.3 (1.1)  n=129 | 4.4 (0.6)  n=129 | 3.6 (1.1)  n=41 | 4.1 (0.7)  n=41 | 3.3 (1.4)  n=18 | 3.9 (0.8)  n=18 | **0.008** |
| Sufficient knowledge of potential barriers to care. | 3.3 (1.1)  n=124 | 4.4 (0.7)  n=124 | 3.5 (1.0)  n=42 | 4.3 (0.6)  n=42 | 2.5 (1.3)  n=17 | 3.8 (0.9)  n=17 | 0.139 |
| **Item asked to community services and health care services only** |  |  |  |  |  |  |  |
| Confidence in own ability to know how and when to inquire about mental health distress. | 3.9 (1.0)  n=126 | 4.2 (0.7)  n=126 | 4.1 (0.7)  n=41 | 4.3 (0.5)  n=41 | n/a | n/a | 0.319 |
| Confidence in my ability to know how and when to inquire about trauma. | 3.6 (1.0)  n=123 | 4.1 (0.7)  n=123 | 3.6 (1.0)  n=42 | 3.9 (1.1)  n=42 | n/a | n/a | 0.312 |
| Sufficient knowledge of how and when to bring up mental health distress and traumatic events. | 3.1 (1.1)  n=126 | 4.0 (0.7)  n=126 | 3.3 (1.0)  n=41 | 4.0 (0.7)  n=41 | n/a | n/a | 0.259 |
| Sufficient knowledge of how cultural and social factors can impact communication. | 3.5 (1.1)  n=127 | 4.4 (0.6)  n=127 | 3.7 (0.9)  n=42 | 4.2 (0.6)  n=42 | n/a | n/a | **0.032** |
| Sufficient knowledge of when to guide/refer children to health care. | 3.3 (1.1)  n=123 | 4.3 (0.8)  n=123 | 4.0 (0.7)  n=42 | 4.3 (0.6)  n=42 | n/a | n/a | **<0.001** |
| Sufficient knowledge of when to guide/refer children to primary-care or child psychiatry. | 3.3 (1.2)  n=124 | 4.3 (0.7)  n=124 | 3.8 (0.9)  n=41 | 4.3 (0.6)  n=41 | n/a | n/a | **0.015** |
| Sufficient knowledge of the collaboration between the municipality and the health care. | 2.7 (1.2)  n=124 | 4.1 (0.8)  n=124 | 3.2 (1.1)  n=41 | 3.9 (0.9)  n=41 | n/a | n/a | **<0.001** |
| Sufficient knowledge of the services offered at the local primary-care based mental health services. | 2.6 (1.2)  n=125 | 4.6 (0.8)  n=125 | 2.8 (1.3)  n=41 | 4.7 (0.5)  N=41 | n/a | n/a | 0.452 |

Measure points: before start of the training program (T0) and just after completion of the training program (T1).

Fully disagree = 1, fully agree = 5

^1^See the supplementary material for full questions.

**Supplementary Table S3.** Mean values and changes in attitudes, knowledge and self-efficacy (T0 and T1), by respondents´ previous experience.

| **Questionnaire item summary (shortened)^1^** | **Experienced^2^** | | **Less experienced^3^** | | **Least experienced^4^** | | **Time* experience** |
| --- | --- | --- | --- | --- | --- | --- | --- |
|  | **T0 mean (Sd)** | **T1 mean (Sd)** | **T0 mean (Sd)** | **T1 mean (Sd)** | **T0 mean (Sd)** | **T1 mean (Sd)** | **p-value** |
| **Item asked to all providers** |  |  |  |  |  |  |  |
| Attitude towards whether it is challenging to work with children and families from migrant backgrounds. | 2.7 (1.4)  n=78 | 2.8 (1.4)  n=78 | 3.1 (1.3)  n=75 | 3.2 (1.2)  n=75 | 2.9 (1.2)  n=25 | 2.8 (1.4)  n=25 | 0.785 |
| Attitude towards whether healthcare in Sweden is equitable. | 3.1 (1.4)  n=67 | 3.4 (1.5)  n=67 | 3.6 (1.5)  n=69 | 3.6 (1.5)  n=69 | 3.4 (1.6)  n=24 | 3.4 (1.3)  n=24 | 0.338 |
| Sufficient knowledge of signs of mental health distress. | 3.4 (1.0)  n=78 | 4.1 (0.7)  n=78 | 3.2 (1.1)  n=78 | 4.0 (0.8)  n=78 | 3.1 (1.2)  n=29 | 4.1 (0.5)  n=29 | 0.355 |
| Sufficient knowledge of how I can work to prevent mental health distress. | 3.2 (1.1)  n=77 | 3.9 (0.7)  n=77 | 3.1 (1.1)  n=78 | 3.9 (0.8)  n=78 | 3.0 (1.3)  n=28 | 3.8 (0.8)  n=28 | 0.766 |
| Sufficient knowledge of how the migration process can impact living conditions. | 3.2 (1.1)  n=77 | 4.3 (0.7)  n=77 | 3.1 (1.2)  n=78 | 4.3 (0.7)  n=78 | 3.0 (1.2)  n=31 | 4.1 (0.9)  n=31 | 0.821 |
| Sufficient knowledge of how the migration process can impact well-being. | 3.3 (1.1)  n=78 | 4.3 (0.7)  n=78 | 3.1 (1.1)  n=78 | 4.3 (0.8)  n=78 | 2.8 (1.2)  n=31 | 4.1 (0.8)  n=31 | 0.370 |
| Sufficient knowledge of how traumatic events can impact mental health. | 3.5 (1.1)  n=78 | 4.3 (0.6)  n=78 | 3.4 (1.1)  n=79 | 4.3 (0.8)  n=79 | 2.9 (1.2)  n=31 | 4.3 (0.6)  n=31 | 0.069 |
| Sufficient knowledge of potential barriers to care. | 3.4 (1.1)  n=77 | 4.3 (0.7)  n=77 | 3.2(1.2)  n=76 | 4.3(0.8)  n=76 | 2.9 (1.2)  n=30 | 4.3 (0.5)  n=30 | 0.113 |
| **Item asked to community services and health care services only** | **T0 mean (Sd)** | **T1 mean (Sd)** | **T0 mean, (Sd)** | **T1 mean, (Sd)** | **T0 mean, (Sd)** | **T1 mean (Sd)** | **Time*experience**  **p-value** |
| Confidence in own ability to know how and when to inquire about mental health distress. | 3.9 (1.0)  n=72 | 4.2 (0.7)  n=72 | 4.0 (0.8)  n=69 | 4.3 (0.6)  n=69 | 4.0 (0.9)  n=26 | 4.3 (0.7)  n=26 | 0.945 |
| Confidence in my ability to know how and when to inquire about traumatic events. | 3.6 (1.0)  n=73 | 4.1 (0.8)  n=73 | 3.6 (1.0)  n=68 | 4.0 (0.8)  n=68 | 3.7 (1.1)  n=24 | 4.0 (0.6)  n=24 | 0.778 |
| Sufficient knowledge of how and when to bring up mental health distress and trauma. | 3.2 (1.0)  n=72 | 4.0 (0.8)  n=72 | 3.1 (1.1)  n=69 | 4.0 (0.8)  n=69 | 3.0 (1.2)  n=26 | 3.9 (0.6)  n=26 | 0.789 |
| Sufficient knowledge of how cultural and social factors can impact communication. | 3.7 (1.0)  n=72 | 4.3 (0.6)  n=72 | 3.6 (1.0)  n=69 | 4.4 (0.7)  n=69 | 3.4 (1.2)  n=28 | 4.4 (0.6)  n=28 | 0.301 |
| Sufficient knowledge of when to guide/refer children with to health care. | 3.4 (1.1)  n=72 | 4.3 (0.8)  n=72 | 3.6 (1.1)  n=66 | 4.3 (0.6)  n=66 | 3.3 (1.1)  n=27 | 4.3 (0.8)  n=27 | 0.498 |
| Sufficient knowledge of when to guide/refer children to primary-care or child psychiatry. | 3.5 (1.2)  n=72 | 4.4 (0.7)  n=72 | 3.5 (1.1)  n=66 | 4.3 (0.6)  n=66 | 3.5 (1.3)  n=27 | 4.3 (0.8)  n=27 | 0.896 |
| Sufficient knowledge of the collaboration between the municipality and the health care. | 2.8 (1.2)  n=71 | 4.1(0.9)  n=71 | 3.0 (1.1)  n=67 | 4.1 (0.8)  n=67 | 2.7 (1.2)  n=27 | 4.0 (0.7)  n=27 | 0.832 |
| Sufficient knowledge of the services offered at the local primary care based mental health services. | 2.7 (1.2)  n=72 | 4.6 (0.7)  n=72 | 2.6 (1.2)  n=67 | 4.6 (0.8)  n=67 | 2.7 (1.3)  n=27 | 4.7 (0.5)  n=27 | 0.842 |

Measure points: before start of the training program (T0) and just after completion of the training program (T1).

Fully disagree = 1, fully agree = 5

^1^ See the supplementary material for full questions.

^2^ Met with children and families from migrant backgrounds > 5 times within the last month.

^3^ Met with children and families from migrant backgrounds 1-5 times within the last month.

^4^ Have not met with children and families from migrant backgrounds within the last month.

**Supplementary Table S4.** Mean values and changes in knowledge (T0 and T1), respondents in the intervention municipality only.

| **Questionnaire item summary (shortened)^1^** | **T0** **mean (Sd)** | **T1 mean (Sd)** | **Mean Diff** | **T0-T1** | |
| --- | --- | --- | --- | --- | --- |
|  |  |  |  | F -value | p-value |
| **Item asked to community services and health care services only** |  |  |  |  |  |
| Sufficient knowledge of the services offered at the local primary-care based mental health services. n=132 | 2.7 (1.2) | 4.6 (0.7) | 1.9 | 286.8 | **<0.001** |
| **Asked to non-profit-organizations only** |  |  |  |  |  |
| Sufficient knowledge of services and support offered at the local primary-care based mental health services. n=8 | 1.8(1.0) | 4.1 (1.4) | 2.4 | 22.8 | **0.002** |

Measure points: before the start of the training program (T0) and just after completion of the training program (T1).

Fully disagree = 1, fully agree = 5

^1^ See the supplementary material for full questions.

**Supplementary Table S5**. Mean values and changes in knowledge (T0, T1, T2) respondents in the intervention municipality only.

| **Questionnaire item summary (shortened)^1^** | **T0 mean (Sd)** | **T1 mean (Sd)** | **T2 mean (Sd)** | **T0-T2** | | **POST HOC T0-T2** | | |
| --- | --- | --- | --- | --- | --- | --- | --- | --- |
|  |  |  |  | F-value | p-value | T0-T1 | T0-T2 | T1-T2 |
|  |  |  |  |  |  | Diff,  p-value | Diff,  p-value | Diff,  p-value |
| **Item asked to community services and health care services only** |  |  |  |  |  |  |  |  |
| Sufficient knowledge of the services offered at the local primary-care based mental health services**.** n=48 | 2.7 (1.3) | 4.8 (0.5) | 4.0 (1.0) | 85.7 | **<0.001*** | 2.0  **<0.001**** | 1.3  **<0.001**** | -0.8  **<0.001**** |

Measure points: before start of the training program (T0), just after completion of the training program (T1), and 3 months after completion of the training (T2).

Fully disagree = 1, fully agree = 5

^1^ See the supplementary material for full questions.

*Sphericity corrected with Greenhouse-Geisser

** All post-hoc p-values corrected with Holm for a family of three

**Supplementary Table S6.** Comparison of participants lost to follow-up and participants retained at follow-up

| Characteristic | Category | Retained at follow-up (n=65) | | Attrition  (n=124) | | Difference |
| --- | --- | --- | --- | --- | --- | --- |
| Target group | | *n* | % | *n* | % | *p* |
|  | Community services | 42 | 65% | 87 | 70% | .64 |
|  | Health care services | 17 | 26% | 25 | 20% |  |
|  | Non-profit organizations | 6 | 9% | 12 | 10% |  |
| Contact with migrant families  (past month) | |  |  |  |  |  |
|  | Yes | 58 | 89% | 99 | 80% | .15 |
|  | No | 7 | 11% | 25 | 20% |  |
| Mental health referrals  (past month) | |  |  |  |  |  |
|  | Yes | 23 | 35% | 45 | 36% | 1.00 |
|  | No | 42 | 65% | 79 | 64% |  |
|  |  |  |  |  |  |  |
|  | | M | SD | M | SD | p |
| Migration related knowledge | | 27.3 | 6.6 | 27.1 | 6.1 | .86 |
| Mental health related knowledge | | 22.1 | 6.2 | 21.9 | 6.1 | .78 |

**Questionnaire items on knowledge, attitudes and self-efficacy^1^**

| Questions asked to all respondents | | Item type |
| --- | --- | --- |
| 1. I find it challenging to work with children and families from migrant backgrounds. | | Attitude |
| 2^2^. I find it interesting to work with children and families from migrant backgrounds. | | Attitude |
| 3. I believe that healthcare for children in Sweden is equitable. | | Attitude |
| 4. I have sufficient knowledge of signs of mental health distress in children. | | Knowledge |
| 5. I have sufficient knowledge of how I can work to prevent mental health distress in children. | | Knowledge |
| 6. I have sufficient knowledge of how the migration process can impact children and families´ living conditions. | | Knowledge |
| 7. I have sufficient knowledge of how the migration process can impact children and families´ psychosocial well-being. | | Knowledge |
| 8. I have sufficient knowledge of how traumatic events can impact children and families´ mental health. | | Knowledge |
| 9. I have sufficient knowledge of potential barriers to care for children and families from migrant backgrounds. | | Knowledge |
| Questions asked to community services and health care only | **Questions asked to non-profit organizations only** |  |
| 10^2^. I understand that the migration process can impact the psychosocial situation of families. | n/a | Attitude |
| 11. I have confidence in my ability to know how and when to inquire about mental health distress when meeting with parents and children. | n/a | Self-efficacy |
| 12. I have confidence in my ability to know how and when to inquire about traumatic events when meeting with parents and children. | n/a | Self-efficacy |
| 13. I have sufficient knowledge of how and when to bring up mental health distress and trauma when meeting with parents and children. | 13^3^. I have sufficient knowledge of how and when to discuss mental health distress with children and families. | Knowledge |
| 14. I have sufficient knowledge of how cultural and social factors can impact communication between professionals and students/clients. | n/a | Knowledge |
| 15. I have sufficient knowledge of when to guide/refer children with mental health distress to health care. | 15^3^. I have sufficient knowledge surrounding where children and young people with mental health distress can go for support within the health care system. | Knowledge |
| 16. I have sufficient knowledge of when to guide/refer children with mental health distress to primary-care or child psychiatry. | n/a | Knowledge |
| 17. I have sufficient knowledge of the collaboration between the municipality and the health care services around children with mental health distress. | n/a | Knowledge |
| 18. I have sufficient knowledge of the services offered at [the local primary care based mental health services] | 18^3^. I have sufficient knowledge surrounding what services and support [the local primary care based mental health services] (child psychologists), can provide. | Knowledge |

n/a = did not receive the question

^1^= translation by the research group

^2^= not analyzed in the study, see the methods section for explanation

^3^ = question reformulated to suit the role of volunteers

^[ ]^ name of the services removed for confidentiality purposes

**Additional information on the study procedure**

In cases where an individual had submitted questionnaires in relation to two separate training tracks (n=7), the first questionnaire to be submitted was analyzed. This was to ensure that the respondents’ answers would not reflect more than one track. In cases where a participant submitted two questionnaires for the same training track (n=2), the first submitted questionnaire was analyzed.
